# Generalization of Knee Joint Moment Prediction During Drop Vertical Jumps Under Graded Visuo-Proprioceptive Conflict: The Role of Multijoint Kinematics Across Validation Frameworks

**DOI:** 10.3390/bioengineering13050524

**Published:** 2026-04-30

**Authors:** Jiarong Wu, Jun Wu, Qiuxia Zhang, Wanli Zang

**Affiliations:** School of Physical Education, Soochow University, Suzhou 215021, China; 20244206013@stu.suda.edu.cn (J.W.); 20235206086@stu.suda.edu.cn (J.W.)

**Keywords:** drop vertical jump, knee joint moment, support vector regression, virtual reality, electromyography, generalization

## Abstract

Accurate estimation of knee joint moment is important for biomechanical monitoring and injury-risk assessment, yet model generalizability under altered sensory environments remains unclear. This study evaluated a support vector regression model for predicting sagittal knee moment during the landing–takeoff cycle of the drop vertical jump (DVJ) under visuo-proprioceptive conflict and examined whether adding hip and ankle kinematics improved performance. Fourteen healthy men performed DVJs under one real and four virtual perturbation conditions with a fixed physical drop height and virtual heights of 0, 10, 30, and 50 cm. Bilateral surface electromyography and three-dimensional lower-limb kinematics were used as inputs, and the inverse-dynamics-derived sagittal knee moment served as the target. Basic and extended feature sets were compared under leave-one-subject-out (LOSO) and leave-one-condition-out (LOCO) frameworks. Within the present experimental design, prediction performance was generally higher under LOCO than under LOSO. Adding hip and ankle kinematics improved prediction mainly under LOCO, whereas gains under LOSO were limited. Waveform similarity showed a non-monotonic decrease-then-recovery pattern across perturbation levels. Residual analysis showed no directional bias, and errors were greater during landing absorption and push-off than during flight. These findings suggest that under the present study design and in this sample, lower performance was observed under LOSO than under LOCO, and that multijoint kinematics may improve prediction robustness under cross-condition settings.

## 1. Introduction

Landing maneuvers are common in ball sports, gymnastics, and military training and impose high mechanical loading on the lower extremities, making them a major mechanism of non-contact anterior cruciate ligament (ACL) injury [1,2,3]. Among the biomechanical variables used to characterize landing-related knee joint loading, the sagittal-plane knee joint moment reflects the net flexion–extension moment output of the joint and is closely associated with anterior tibial shear force and ACL strain during the landing deceleration phase; it is therefore regarded as a key indicator in injury risk assessment and real-time biofeedback [3,4]. Accurate and continuous estimation of this variable during dynamic tasks may provide a methodological basis for future wearable monitoring, clinical decision support, and on-field injury surveillance [5,6,7,8].

Knee joint moments are obtained primarily using inverse dynamics [9]. This method calculates the net joint moment within a rigid-body mechanical model by integrating synchronized motion capture and force plate data and is widely regarded as a reference standard. However, this approach relies heavily on laboratory-grade equipment, typically requiring optical motion capture systems and floor-embedded force plates, which limits its application in field monitoring, clinical assessment, and long-term training surveillance [9]. With the development of wearable sensor technology, constructing predictive models from more accessible signals, such as surface electromyography (sEMG) and kinematics, to reduce dependence on laboratory infrastructure has become an important direction in knee joint moment estimation [10].

Machine learning approaches have demonstrated the feasibility of predicting lower-limb joint moments from electromyography and kinematic signals during tasks such as gait, squatting, and stair ascent and descent, but the available evidence has been derived mainly from relatively low-speed or cyclic tasks, and its applicability to high-impact landing tasks remains to be further established. Among these approaches, support vector regression (SVR) is attractive for small-sample biomechanical settings because it provides a favorable bias–variance balance. This makes SVR a reasonable choice when the primary goal is to examine generalization patterns and input-feature effects under limited-sample biomechanical conditions, rather than to benchmark algorithmic superiority across multiple model families. Nevertheless, two key gaps remain in the existing literature [11,12]. First, regarding feature design, prior studies have largely used target-joint-related local information as inputs, such as peri-knee muscle electromyography and knee joint kinematics, while relatively few have systematically examined the independent contribution of upstream and downstream joint kinematics, such as those of the hip and ankle, to knee joint moment prediction [13]. From a kinetic-chain perspective, the lower extremity is not a simple sum of isolated joints, but rather a system in which knee loading is jointly determined through intersegmental energy transfer and multi-joint coordination. Therefore, whether adding hip and ankle kinematics can consistently improve the accuracy of knee joint moment prediction still lacks rigorous comparison [14]. Second, regarding generalization assessment, many studies have reported overall predictive accuracy without explicitly distinguishing between two fundamentally different generalization demands, namely cross-subject generalization and cross-task-condition generalization. The former is influenced mainly by inter-individual neuromuscular differences, whereas the latter more strongly reflects biomechanical adaptation induced by changes in task conditions. Conflating the two may obscure the model’s practical performance boundaries [15].

The ecological validity of the model is further challenged by the complexity of the sensory environment in real-world movement contexts. Visual information, particularly optic flow cues related to descent height, can markedly modulate pre-landing muscle pre-activation, joint stiffness control, and impact absorption strategies [16,17,18,19]. In natural movement settings, athletes often encounter different heights, spatial references, and visual environments; accordingly, a model trained under a single standardized laboratory condition may not generalize reliably to tasks in which the visual–motor context changes. Virtual reality (VR) provides a controllable experimental platform for testing this question. By manipulating virtual drop height while keeping the true mechanical boundary conditions unchanged, VR can systematically induce graded visuo-proprioceptive conflict, thereby allowing examination of whether the knee joint moment mapping learned from electromyography and kinematic signals remains stable across different levels of sensory perturbation [20]. To date, it remains unclear whether visually induced sensory conflict affects the predictive performance of knee joint moment estimation or whether this mapping retains a certain degree of robustness.

The present study aimed to systematically evaluate the generalization performance of an SVR-based knee flexion–extension moment prediction model during the first landing–takeoff cycle of the drop vertical jump (DVJ) under graded visuo-proprioceptive conflict. Specifically, 14 healthy male participants performed DVJs under one real condition and four virtual height perturbation conditions. Bilateral lower-limb sEMG and three-dimensional kinematics were used as inputs, and the inverse-dynamics-derived sagittal knee joint moment served as the prediction target. Two input configurations were compared: a basic feature set composed of multi-muscle sEMG and knee joint kinematics, and a multijoint feature set that further incorporated hip and ankle joint angles and angular velocities. In addition, leave-one-subject-out (LOSO) and leave-one-condition-out (LOCO) frameworks were used to evaluate cross-subject and cross-condition generalization, respectively. Specifically, this study addressed three questions: first, how model performance differed between the two evaluation frameworks, representing cross-subject and cross-condition generalization, under the present study design; second, whether adding hip and ankle kinematics can systematically improve predictive performance, and whether this improvement depends on the generalization scenario; and third, whether the magnitude of visual perturbation systematically affects predictive accuracy and error structure.

## 2. Materials and Methods

### 2.1. Participants and Experimental Procedures

This study recruited 14 healthy male participants from a school of physical education who had no known neurological disorders and no recent lower-limb injuries (age: 22.14 ± 2.54 years; body mass: 73.51 ± 8.32 kg; height: 177.44 ± 5.85 cm; see Table A1 for details). All experimental procedures conformed to the principles of the Declaration of Helsinki and were approved by the Ethics Committee of Soochow University (approval number: SUDA20240430H12).

**Figure 1:**
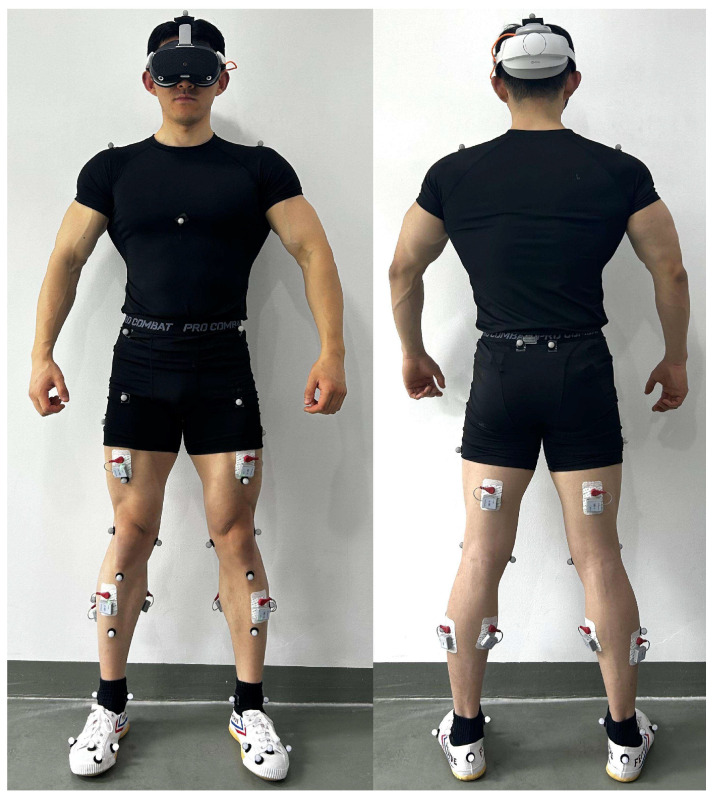
Reflective marker locations and sEMG electrode placement.

Kinematic data were collected at 100 Hz using a three-dimensional motion capture system composed of 16 infrared cameras (MX13, Vicon Motion Systems, (MX13; Vicon Motion Systems Ltd., Oxford, UK), with 38 reflective markers placed according to the Plug-in-Gait model. Kinetic data were collected at 1000 Hz using two force plates embedded in the ground (9281EA; Kistler Instrumente AG, Winterthur, Switzerland). sEMG signals from six target muscles on both sides (rectus femoris, biceps femoris, tibialis anterior, gastrocnemius, soleus, and gluteus maximus) were synchronously recorded at 1000 Hz using a wireless EMG system (Zhiyunwei, Shanghai, China) (Figure 1). All devices were time-synchronized via an MX Ultranet HD unit.

The experimental task was the DVJ: participants stepped off a 30 cm box onto the force plates with both feet and then immediately performed a maximal vertical jump, with their hands kept on their hips throughout. Five conditions were tested, including one real-environment condition (30 cm, without a VR device) and four virtual visual perturbation conditions. Under the virtual conditions, participants wore a head-mounted display (HMD; PICO 4 Pro; Pico Technology Co., Ltd., Beijing, China); the actual drop height remained unchanged, whereas the virtual height was set at 0, 10, 30, and 50 cm. The order of conditions was randomized, and 3–4 valid trials were recorded for each condition; a valid trial was defined as one in which both feet landed fully on the force plates and no marker was lost. The dominant limb was determined by self-report as the limb habitually used for kicking a ball or taking off in a single-leg jump in daily life.

### 2.2. Data Processing and Phase Segmentation

EMG signals were band-pass filtered at 20–450 Hz, followed offline by direct current offset removal and full-wave rectification. Root mean square (RMS) values were then calculated using a 100 ms sliding window with a 50 ms step size and used as EMG features. Kinematic and kinetic data were processed in Visual3D (version 2023.08.3; C-Motion, Inc., Germantown, MD, USA). Three-dimensional joint angles and angular velocities of the hip, knee, and ankle were calculated using a Cardan rotation sequence (X–Y–Z), and the sagittal-plane knee joint moment was computed using inverse dynamics and normalized to body mass (N·m/kg). The processed signals were then low-pass filtered using a fourth-order zero-phase Butterworth filter at 6 Hz and time-normalized to 101 points (0–100%) over the movement cycle.

The full DVJ task was divided into eight consecutive functional phases (Phase 1–8) using a multi-signal integrated identification algorithm (see Table A2). This study focused on the first landing–takeoff sequence, with Phase 3 (aerial descent), Phase 4 (initial landing absorption), Phase 5 (push-off), and Phase 6 (aerial flight) selected as the main analysis period.

### 2.3. Input Feature Composition

This study compared two input feature configurations. Input 1 (12 dimensions) consisted of EMG RMS values from six target muscles, together with three-axis knee joint angles (×3) and angular velocities (×3). On this basis, Input 2 (24 dimensions) further included hip and ankle joint angles (×3 each) and angular velocities (×3 each), forming a feature set that covered a more complete lower-limb kinetic chain. The EMG features were identical in the two configurations, and the only difference lay in the range of kinematic information, thereby allowing evaluation of the combined contribution of added hip and ankle kinematics to knee joint moment prediction. All features were z-score standardized before being entered into the model. To prevent data leakage, standardization parameters were estimated using the training set only.

### 2.4. Prediction Model

This study used SVR to predict the knee flexion–extension moment at each time point during the landing task [21,22]. By constructing a regression decision function in a high-dimensional feature space, SVR offers certain advantages for high-dimensional, small-sample biomechanical data. Accordingly, SVR was selected to provide a stable nonlinear regression framework for the present analyses. The model used a radial basis function (RBF) kernel to accommodate the nonlinear mapping between EMG signals and joint moments. The epsilon-insensitive loss parameter was set to 0.01 to balance predictive accuracy and model complexity.

Model hyperparameters were optimized using a nested grid search strategy. Candidate values for the regularization parameter C were {0.1, 1, 10, 100}, and candidate values for the kernel width parameter gamma were {‘scale’, 0.01, 0.1, 1.0}, yielding 16 hyperparameter combinations in total. The inner cross-validation used 3-fold KFold (random seed fixed at 42), with negative root mean square error (neg_RMSE) as the optimization objective, and the optimal hyperparameter combination was identified through parallel GridSearchCV. Data used in the inner cross-validation were strictly confined to the training set of the current iteration and were fully isolated from the test set [23].

### 2.5. Data Partitioning and Generalization Evaluation Frameworks

EMG features from corresponding muscles and joint kinematic features from the left and right trials were mapped onto the same feature dimensions (e.g., the RMS of the left and right rectus femoris both corresponded to Dimension 1), thereby allowing bilateral data to be unified within the same feature space and then combined for model training and prediction. Dominant-versus-non-dominant comparisons were performed as a methodological check of potential side-specific bias rather than as a primary study objective. On this basis, this study adopted two complementary generalization evaluation frameworks.

Scene 1—LOSO: with the subject as the held-out unit, all trials (including both limbs) from one subject were used as the test set in each round, and data from all remaining subjects were used as the training set, until each subject had been tested once. The full LOSO procedure was conducted independently for each of the five experimental conditions to evaluate the model’s cross-subject generalization ability for unseen subjects [13]. Under this framework, the training set in each round contained trials from 13 subjects under a single experimental condition, and no data from the test subject were included in training, ensuring a strict evaluation of cross-subject generalization.

Scene 2—LOCO: with the experimental condition as the held-out unit, all trials from one condition across all subjects were used as the test set in each round, and data from the other four conditions were combined as the training set, for a total of five rounds. This framework evaluated the model’s cross-task generalization ability for unseen landing conditions. Under this framework, the training set in each round contained trials from all 14 subjects under four conditions, making the training set approximately four times larger than that under the LOSO framework; at the same time, the neuromuscular and kinematic data of the test subjects under the other four conditions were already included in the training set. These two aspects are structural characteristics of the LOCO framework arising from its design principle of using the condition as the held-out unit and should therefore be fully considered when interpreting performance differences between the two frameworks.

### 2.6. Performance Evaluation and Statistical Analysis

Model predictive performance was evaluated using the normalized root mean square error (NRMSE), which is defined as:

$$NRMSE=\frac{\sqrt{\frac{1}{N}\sum_{t=1}^{N}(y_{t}-{\hat{y}}_{t}{)}^{2}}}{y_{max}-y_{min}}\times 100\%$$

where $y_{t}$ denotes the true moment value, ${\hat{y}}_{t}$ denotes the predicted moment value, and $y_{max}-y_{min}$ denotes the value range of the true moment. NRMSE standardizes the error by the amplitude range of the true signal, thereby eliminating the influence of inter-subject differences in moment magnitude; lower values indicate better predictive accuracy.

On this basis, further analyses were conducted from three perspectives. All inferential tests were performed at the subject level, whereas trial-level values were used for descriptive visualization only. Differences in predictive performance between Input 1 and Input 2 were evaluated using subject-level paired Wilcoxon signed-rank tests. For Table 1 metrics (NRMSE and R^2^), Bonferroni correction was applied across 10 comparisons within each scene (5 conditions × 2 metrics; corrected α = 0.005). For waveform similarity (Pearson correlation coefficient, r), Bonferroni correction was applied across five condition-wise comparisons within each scene (corrected α = 0.010). For residual time-series analysis, the difference between the true and predicted values (residual = true value − predicted value) was evaluated at the overall level using one-sample Wilcoxon signed-rank tests (H_0_: median residual = 0) to assess systematic bias, with Bonferroni correction applied across the five experimental conditions within each scene–input configuration (corrected α = 0.010); effect size was reported as the rank-biserial correlation (rb), with |rb| ≥ 0.50 defined as a large effect. At the time-series level, participant-level 95% confidence intervals were calculated at each time point, and a systematic bias interval was defined when the CI excluded zero for five or more consecutive time points. For phase-specific prediction accuracy analysis, segmental NRMSE for the landing absorption plus push-off period (Phase 4–5) and the aerial flight period (Phase 6) was normalized by the full-waveform amplitude range, and Wilcoxon signed-rank tests were used to compare phase-specific predictive differences between the dominant and non-dominant limbs (40 paired comparisons; Bonferroni-corrected α = 0.00125).

## 3. Results

### 3.1. Lower-Limb Kinematics and Knee Joint Moment Prediction Accuracy

**Figure 2:**
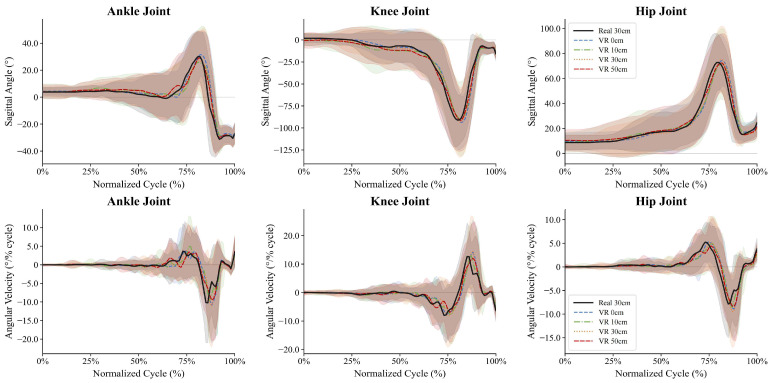
Sagittal plane joint angles and angular velocities of the ankle, knee, and hip during the first landing cycle of the drop vertical jump under five visual conditions. Note. Group-mean sagittal plane joint angles (°) of the ankle (**left**), knee (**middle**), and hip (**right**) across the normalized first landing cycle of the DVJ (0–100%), spanning from the aerial descent phase to the subsequent takeoff. Solid lines represent condition means; shaded bands indicate ±1 SD.

As shown in Figure 2, the sagittal-plane kinematics of the three joints during the first landing cycle of the DVJ exhibited a typical biphasic landing–takeoff pattern: joint angles remained stable during the aerial phase, flexed rapidly during the landing absorption phase (with peak knee angles of approximately 90–100° and hip angles of approximately 70–75°, while the ankle exhibited a biphasic change from dorsiflexion to plantarflexion), and then declined during the push-off phase, with the angular velocity curves showing corresponding directional reversals.

The comparison of predictive performance between the two input feature configurations is presented in Table 1. Overall, lower NRMSE values and higher R^2^ values were observed under the LOCO framework than under the LOSO framework within the present study design.

Under the cross-subject generalization framework (Scene 1), Input 2 outperformed Input 1 only in the VR 10 cm condition for both NRMSE (0.128 ± 0.027 vs. 0.154 ± 0.027, *p* < 0.001) and R^2^ (0.621 ± 0.143 vs. 0.459 ± 0.166, *p* < 0.001), whereas no statistically significant differences were observed in the other four conditions.

Under the cross-condition generalization framework (Scene 2), reductions in NRMSE for Input 2 were observed across all five conditions (30 cm: *p* = 0.0081; VR 0 cm: *p* < 0.001; VR 10 cm: *p* < 0.001; VR 30 cm: *p* = 0.0061; VR 50 cm: *p* = 0.0046). Using the Bonferroni-corrected threshold for Table 1 metrics (corrected α = 0.005), significant NRMSE reductions were observed in three conditions (VR 0 cm, VR 10 cm, and VR 50 cm), whereas the 30 cm and VR 30 cm conditions did not survive correction. For R^2^, significant improvements were observed in the VR 0 cm and VR 10 cm conditions (both *p* < 0.001), whereas the remaining conditions did not survive Bonferroni correction.

The inclusion of full lower-limb kinematics (Input 2) produced a more robust improvement in the prediction of knee joint moment amplitude under the cross-condition generalization framework, suggesting that hip and ankle kinematic information provides important complementary value when the model generalizes to unseen landing conditions.

### 3.2. Waveform Similarity of Knee Joint Moment Prediction

**Figure 3:**
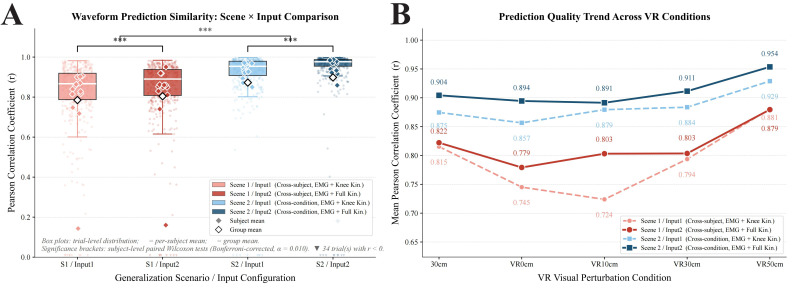
Waveform prediction similarity across scene–input combinations and VR conditions. (**A**) Trial-level Pearson correlation distributions across the four scene–input combinations. Box plots and dots represent trial-level r values (one value per valid trial). Open diamonds indicate subject-level means (mean of all valid trials for that subject within each scene × input × condition cell), and filled diamonds indicate group means. Statistical comparisons (Input 1 vs. Input 2) were performed using paired Wilcoxon signed-rank tests on subject-level mean r values (one paired observation per subject per condition). Bonferroni correction was applied within each scene across five condition-wise comparisons (corrected α = 0.010). (**B**) Condition-level mean Pearson correlation coefficient across the five VR perturbation conditions for each scene–input combination. *** *p* < 0.001.

As shown in Figure 3A, numerical differences in waveform prediction quality were observed between the two generalization scenes: the subject-level mean Pearson correlation coefficient (r) in the cross-condition generalization scene (Scene 2) was 0.884 ± 0.189 for Input 1 and 0.911 ± 0.197 for Input 2, approximately 0.09 correlation units higher than that in the cross-subject generalization scene (Scene 1; r = 0.791 ± 0.189 and 0.817 ± 0.181); at the same time, the medians in Scene 1 (r = 0.847 and 0.855) were markedly higher than the corresponding means, whereas the medians in Scene 2 (0.944 and 0.971) were at a higher level.

In terms of input configuration, the pattern of improvement in prediction quality with Input 2 was not identical across the two generalization scenes: in Scene 2, paired Wilcoxon signed-rank tests showed that Input 2 reached Bonferroni-corrected significance in 3 of the 5 experimental conditions (the correction was based on 5 paired comparisons for 5 conditions × 1 metric; corrected α = 0.010) (30 cm: Δr = +0.030, *p* < 0.001, rb = 1.000; VR 30 cm: Δr = +0.028, *p* = 0.006, rb = 0.824; VR 50 cm: Δr = +0.025, *p* = 0.001, rb = 0.934). In the remaining 2 conditions (VR 0 cm: Δr = +0.038, rb = 0.752; VR 10 cm: Δr = +0.012, rb = 0.714), although corrected significance was not reached, effect sizes were large in both cases, and the corresponding NRMSE differences had already reached Bonferroni-corrected significance in Section 3.1 (both *p* < 0.001), whereas the corresponding Pearson r values did not reach corrected significance. In Scene 1, by contrast, corrected significance was observed only in the VR 10 cm condition (Δr = +0.079, *p* < 0.001, rb = 1.000), whereas improvements in the other 4 conditions were very limited (Δr = −0.001 to +0.034, all *p* ≥ 0.241). In the VR 50 cm condition, the waveform similarity of Input 2 was even slightly lower than that of Input 1 (Δr = −0.001, rb = −0.011), and the overall gain was smaller than that in Scene 2.

In addition, a small number of extreme failure trials with r < 0 were observed in each configuration (approximately 2% in each combination), corresponding to directional reversal between the predicted and measured waveforms. These trials are marked at zero with ▼ in Figure 3A and were fully included in the statistical analysis. Complete paired test statistics comparing Input 2 and Input 1 under each VR condition are provided in Table A3. It should also be noted that the conditions in which significant advantages of Input 2 were detected differed systematically between NRMSE and Pearson r under the Scene 2 framework: NRMSE reached corrected significance in the VR 0 cm, VR 10 cm, and VR 50 cm conditions, whereas Pearson r reached corrected significance in the 30 cm, VR 30 cm, and VR 50 cm conditions, with VR 50 cm being the only condition that passed Bonferroni correction for both metrics.

As shown in Figure 3B, all four combinations exhibited a non-monotonic “decrease-then-recovery” pattern under the VR conditions: prediction quality declined to varying degrees during low-to-moderate perturbation levels (VR 0 cm–VR 10 cm) (minimum values: Scene 1/Input 1, r = 0.724; Scene 2/Input 2, r = 0.891), then increased continuously with increasing perturbation magnitude, reaching the highest prediction quality in the VR 50 cm condition for all combinations (Scene 1/Input 1: r = 0.881; Scene 1/Input 2: r = 0.879; Scene 2/Input 1: r = 0.929; Scene 2/Input 2: r = 0.954), showing a pattern of initial decline followed by recovery.

### 3.3. Residual Time-Series Analysis of Knee Joint Moment Prediction

**Figure 4:**
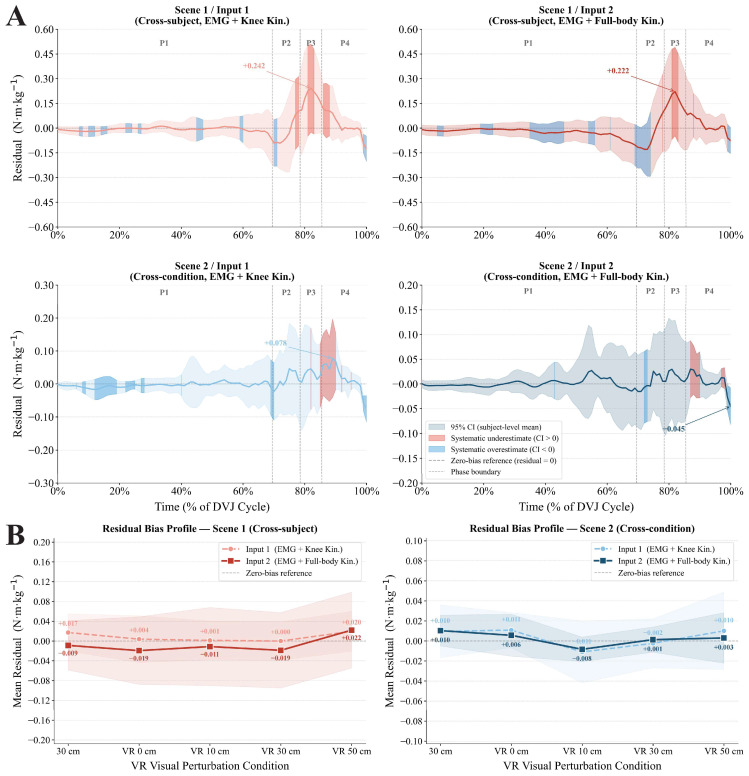
Residual time-series and condition-level residual bias across scene–input combinations. (**A**) Time-series residual profiles across the DVJ cycle for all scene–input combinations. (**B**) Condition-level mean residual bias profiles across the five VR perturbation conditions. Note. Residual = y_true − y_pred (positive: underestimation; negative: overestimation). Shaded bands = participant-level 95% CI. Colored overlays = systematic bias zones (red: underestimation; blue: overestimation; criterion: CI excludes zero for ≥ 5 consecutive time points). Vertical dashed lines indicate phase boundaries in panel A. Phase labels denote functional movement phases: P1 = Free Fall, P2 = Initial Landing, P3 = Push-off, and P4 = Flight. Annotated values = signed peak residuals (N·m·kg^−1^).

In Scene 1, the time-series residual curves (upper row of Figure 4A) remained close to zero overall during the stance and aerial phases of the DVJ, with only transient peaks appearing during the landing deceleration phase (75–90%) (Input 1: +0.242 N·m·kg^−1^; Input 2: +0.222 N·m·kg^−1^); the confidence intervals at the peak locations all crossed zero, and no evidence of systematic bias was observed. In Scene 2, the amplitude of the residual curves was markedly reduced (lower row of Figure 4A), with time-series peaks reaching only 32% (Input 1) and 20% (Input 2) of those in Scene 1, and the overall residual amplitude was lower than that in Scene 1.

The residual bias profiles (Figure 4B) showed that the condition-level mean residuals in both scenes did not increase monotonically with VR perturbation intensity (0–50 cm), indicating that the systematic directional bias of prediction residuals did not increase monotonically with visual perturbation magnitude, and no fixed directional pattern was observed across perturbation conditions. As shown by the statistical results in Table 2, after Bonferroni correction across the five experimental conditions within each scene–input configuration (corrected α = 0.010), none of the 20 condition-level one-sample Wilcoxon signed-rank tests reached significance (all corrected *p* > 0.010), and no stable directional bias in prediction errors was observed across perturbation magnitudes. It is worth noting that, in Scene 2, two tests showed large effect sizes (Input 1 VR 0 cm: rb = +0.619, *p* = 0.042; Input 2 VR 10 cm: rb = −0.692, *p* = 0.027), although neither remained significant after Bonferroni correction.

### 3.4. Limb Dominance Differences in Prediction Accuracy During the Landing Phase

The first landing-related period of the DVJ was further divided into the landing absorption plus push-off phase and the aerial flight phase, and segmental NRMSE normalized by the full-waveform amplitude range was used to evaluate phase-specific prediction errors.

**Figure 5:**
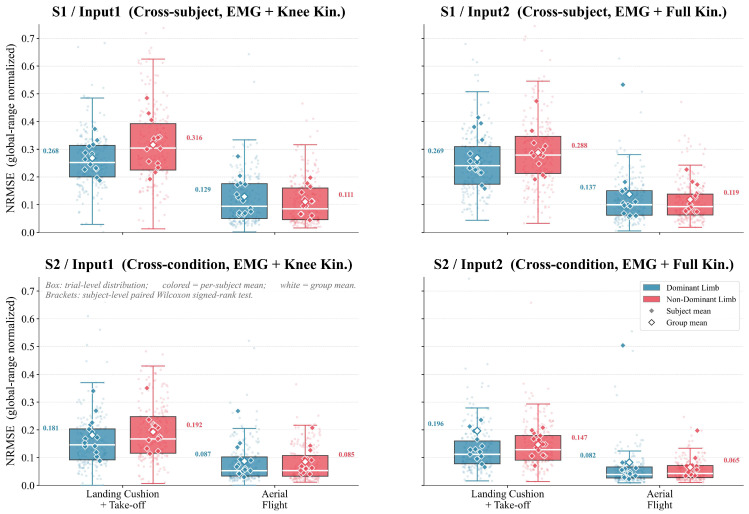
Phase-specific NRMSE distributions by limb dominance across scene–input combinations.

As shown in Figure 5, prediction errors during the landing absorption plus push-off phase were consistently higher than those during the aerial flight phase across all scene–input combinations. In Scene 1, mean NRMSE during the landing phase ranged from 0.268 to 0.316, approximately 2.1–2.4 times that during the aerial flight phase (0.111–0.137); in Scene 2, this difference was maintained (landing: 0.147–0.196; flight: 0.065–0.087).

In terms of limb dominance, no stable difference in prediction accuracy was observed between the dominant and non-dominant limbs. Paired Wilcoxon signed-rank tests were performed on subject-level mean segmental NRMSE values (one paired observation per subject per scene × input × condition × phase). Of the 40 paired comparisons, 6 reached nominal significance (all *p* < 0.05), but the directions were inconsistent; after Bonferroni correction for 40 comparisons (corrected α = 0.00125), only one comparison remained significant. Moreover, all absolute between-limb differences were below 0.025 NRMSE, indicating limited practical side-related disparity in prediction error.

## 4. Discussion

This study examined the performance of an SVR-based knee joint moment prediction model under different generalization frameworks in the presence of graded visuo-proprioceptive conflict and evaluated whether the contribution of multijoint kinematic information depended on the generalization context. The results showed that, under the present study design, prediction performance was generally better under the cross-condition framework than under the cross-subject framework, as reflected by lower amplitude error and higher waveform similarity. The predictive benefit of multijoint kinematic features was observed primarily under the cross-condition framework rather than as a consistent advantage across frameworks. The effect of visual perturbation on prediction quality was non-monotonic, and prediction errors were concentrated in the landing absorption and push-off phases. This study helps clarify the generalization boundaries of knee joint moment prediction under visually perturbed conditions.

The lower performance observed under the cross-subject framework suggests that, within the present study design, transferring the model to unseen individuals was more challenging than transferring it to unseen conditions. Compared with the cross-condition framework, the LOSO approach requires the model to transfer a learned predictive relationship to a new individual without exposure to any data from that target participant. The lower performance under this framework may therefore reflect both the smaller training set and greater deviation of the input–output relationship from the training data distribution when the model is applied to unseen individuals [24,25,26]. Furthermore, waveform similarity results showed that the decline was not uniform across all samples, but was driven mainly by a small subset of individuals with clearly lower prediction quality. This pattern suggests that cross-subject prediction failure may manifest as a pronounced mismatch in some individuals rather than as a mild decline distributed evenly across the sample. This observation is broadly consistent with prior joint torque prediction studies, which have shown that cross-subject prediction is often more difficult than prediction in previously observed individuals, in part because of inter-individual parameter differences, out-of-distribution samples, and limited training data [27,28,29]. In this study, these two sources of generalization challenge were examined within the same DVJ and visual perturbation platform, allowing their performance patterns to be compared within a common task context. It should be noted that, in the present study, the cross-condition framework had advantages in both training sample size and information content; therefore, the performance difference between the two frameworks cannot be attributed entirely to differences in the intrinsic difficulty of individual variation and condition switching.

The predictive value of multijoint kinematic features was scenario-dependent. The present results showed that, after the inclusion of hip and ankle joint angles and angular velocities, improvements in model performance were observed mainly under the cross-condition framework, whereas gains under the cross-subject framework were relatively limited. This suggests that their value may lie less in increasing feature number than in providing information beyond knee-local features when task conditions change [27,30]. For the same participant, although visual perturbation may alter landing strategy, multijoint kinematic features may still retain information useful for cross-condition transfer, thereby improving the model’s ability to transfer to unseen conditions [16,18]. By contrast, when the generalization target shifts to unseen individuals, the added hip and ankle kinematics are insufficient to compensate for structural differences in the EMG–moment mapping at the individual level, and the resulting predictive gain is therefore constrained. Previous studies have suggested that additional biomechanical information or physical priors can improve joint torque prediction, particularly under conditions of high generalization pressure [27,30]. The present findings are consistent with this line of research, but suggest that such gains depend on the dominant type of generalization challenge. In addition, NRMSE and Pearson’s r showed different significance patterns in some conditions. The former mainly reflects moment amplitude error, whereas the latter places greater emphasis on the reproduction of waveform temporal shape; accordingly, multijoint kinematics may preferentially improve different aspects of prediction quality under different conditions. These findings suggest that multijoint kinematics may contribute differently to amplitude estimation and waveform reproduction, and that their predictive value depends on the generalization context.

The non-monotonic pattern of the visual perturbation effect was a key finding. Previous studies on visual information and landing control have shown that visual height perception, visual occlusion, and related sensory cues affect pre-landing muscle activation, joint stiffness regulation, and post-landing biomechanical performance [16,18,19]. Against this background, the present study was concerned not with whether visual perturbation alters landing control itself, but with how such alterations further influence the performance of joint moment prediction models. Model performance would conventionally be expected to decline as visuo-proprioceptive conflict increases; however, no such linear trend was observed. Instead, across different input configurations and generalization frameworks, prediction quality showed a decline at moderate perturbation levels followed by recovery at higher perturbation levels. The relatively poorer performance observed around VR 10 should therefore be interpreted as part of this broader low-to-moderate perturbation dip, rather than as a uniformly isolated condition-specific effect. This result suggests that the effect of visual perturbation on knee joint moment prediction may not vary as a simple linear function of conflict intensity. One tentative interpretation is that changes in sensory–motor coupling stability under perturbed conditions may have contributed to the observed decrease-then-recovery pattern, although this mechanism was not directly examined in the present study and should therefore be regarded as hypothesis-generating rather than as a mechanistic inference [20]. Residual analysis further informs this pattern: although prediction quality fluctuated across perturbation conditions, no increase in systematic directional bias was observed with increasing perturbation magnitude, suggesting that the performance changes more likely reflect altered prediction dispersion rather than sustained directional overestimation or underestimation.

Across movement phases, the current model’s prediction difficulty was concentrated mainly in the initial landing absorption phase and the subsequent push-off phase, rather than being evenly distributed across the DVJ cycle. This finding is consistent with prior biomechanical evidence identifying the landing absorption phase as a critical window in which lower-limb loading changes most rapidly and control demands are greatest [31,32]. Under both the cross-subject and cross-condition frameworks, and with both local and multijoint inputs, errors during landing-related phases were consistently higher than those during the aerial flight phase, indicating that these phases were a common source of difficulty rather than an incidental pattern under a specific generalization scenario or input configuration. For an SVR model based on point-by-point regression, this pattern is biomechanically plausible. Although the model can establish a nonlinear mapping from the current EMG and kinematic inputs to joint moment, it lacks explicit temporal memory and cannot use recent trajectory information during the rapid rise in joint moment. In addition, pre-landing muscle pre-activation typically precedes the mechanical response [16,33], whereas RMS features extracted with a sliding window may smooth this temporal relationship. As a result, temporal mismatch may exist between the neuromuscular state reflected by the inputs and the target joint moment value; the transient residual peak observed during the landing deceleration phase under the cross-subject framework is consistent with this interpretation, although it does not constitute direct proof. From a practical perspective, this phase-specific error structure suggests that the current model may be more suitable for monitoring overall waveform trends or relative changes across conditions than for applications requiring precise estimation during the most mechanically demanding landing-related phases. Because landing absorption and push-off are also the phases most relevant to rapid load transfer and potential injury-related interpretation, error concentrated in these periods may be more consequential for screening or real-time feedback than comparable error during flight. By contrast, limb-side analysis showed no systematic difference between the dominant and non-dominant limbs, indicating that prediction errors did not exhibit a stable side-specific bias under the current sample and task conditions and supporting, from a methodological perspective, the feasibility of mapping bilateral data into the same feature space and modeling them jointly. Under the current sample size, bilateral pooling also increases the amount of available training information without introducing evidence of systematic side-specific bias. Further improvement in model performance may depend on introducing sequence models capable of capturing temporal dependence, thereby more effectively characterizing the rapidly evolving neuromuscular–moment coupling during landing absorption [10].

Although the present study offers preliminary evidence regarding the generalization boundaries of knee joint moment prediction under visually perturbed conditions, several methodological limitations should be considered when interpreting the findings. First, the participants were limited to a small and homogeneous sample of healthy young men; therefore, the current conclusions cannot be directly generalized to women, adolescents, populations at high risk of sports injury, or clinical individuals with neuromuscular dysfunction. Given that these populations may differ systematically in landing control, joint stiffness regulation, and sensory integration, future studies are needed to test the applicability of the present findings in broader and more diverse samples. Second, the LOSO and LOCO frameworks differ structurally in both training sample size and whether partial information from the test individual is included; therefore, the performance gap between them cannot be attributed entirely to generalization difficulty itself. Future work should further distinguish the effects of inter-individual variation from those of data scale through designs with stricter control of training information volume. Third, although the present study was motivated in part by the long-term goal of deployable prediction, data collection still relied on laboratory-grade motion capture, force plates, and synchronized EMG systems. Accordingly, the current findings are more informative with respect to generalization mechanisms than to the feasibility of field deployment. To move the model into real training or clinical environments, future studies should verify whether similar generalization patterns can be retained when key kinematic features are reconstructed from IMUs or other simplified sensing schemes. In addition, the present model used SVR for point-by-point prediction. Although this choice helps maintain model stability under small-sample conditions, it also limits the ability to capture the rapidly time-varying dynamics of the landing absorption phase. We also did not perform an ablation analysis to separate the individual contributions of hip versus ankle kinematics, nor did we evaluate broader feature sets such as frequency-domain EMG descriptors or joint acceleration. Therefore, the present findings should be interpreted as evidence for the combined value of the expanded multijoint feature set rather than for the specific contribution of any single added feature group. Future studies may compare models with explicit temporal memory, such as LSTM, TCN, or Transformer architectures, to determine whether they can mitigate error accumulation during the landing phase. Finally, given the lower performance observed under the cross-subject framework in the present study, future methodological optimization may need to move beyond a single global generic model and instead explore individualized adaptation strategies based on a small amount of target-subject data, such as transfer learning or lightweight recalibration frameworks, in order to reduce performance loss when transferring the model from observed populations to new individuals. Overall, despite these limitations, the present study offers a useful starting point for understanding the generalization boundaries of knee joint moment prediction under visually perturbed conditions and offers directions for future optimization centered on individualized adaptation, simplified sensor configurations, and temporal modeling.

## 5. Conclusions

This study examined the generalization performance of an SVR-based knee flexion–extension moment prediction model during the DVJ landing task under graded visuo-proprioceptive conflict in healthy young men. The results showed that prediction performance was generally lower under the cross-subject framework than under the cross-condition framework under the present study design. The predictive benefit of multijoint kinematic features was observed mainly under the cross-condition framework and did not emerge as a consistently stable advantage. The effect of visual perturbation on prediction quality followed a non-monotonic pattern, with performance declining at moderate perturbation levels and partially recovering at higher perturbation levels; phase analysis further showed that prediction errors were concentrated in the landing absorption and push-off phases. This study helps clarify the generalization boundaries of knee joint moment prediction under visually perturbed conditions in healthy young men and provides a starting point for future work on individualized adaptation, multijoint information integration, and temporal modeling.

## Figures and Tables

**Table 1:** Scene 1 (LOSO) and Scene 2 (LOCO)—Input 1 (12-dim) vs. Input 2 (24-dim): Prediction Performance (Bonferroni-corrected Wilcoxon Signed-Rank Test).

	NRMSE			R^2^		
Condition	Input 1 (Mean ± SD)	Input 2 (Mean ± SD)	*p*	Input 1 (Mean ± SD)	Input 2 (Mean ± SD)	*p*
Scene 1—Cross-subject Generalization (LOSO)						
30 cm	0.117 ± 0.023	0.117 ± 0.029	0.8394 ns	0.677 ± 0.121	0.678 ± 0.126	0.9460 ns
VR 0 cm	0.125 ± 0.036	0.121 ± 0.025	0.9032 ns	0.537 ± 0.253	0.582 ± 0.143	0.9032 ns
VR 10 cm	0.154 ± 0.027	0.128 ± 0.027	<0.001 ***	0.459 ± 0.166	0.621 ± 0.143	<0.001 ***
VR 30 cm	0.121 ± 0.027	0.115 ± 0.034	0.2439 ns	0.650 ± 0.094	0.688 ± 0.100	0.2163 ns
VR 50 cm	0.115 ± 0.021	0.116 ± 0.020	0.3054 ns	0.689 ± 0.144	0.695 ± 0.089	0.3396 ns
Scene 2—Cross-condition Generalization (LOCO)						
30 cm	0.082 ± 0.028	0.067 ± 0.033	0.0081 ns	0.833 ± 0.102	0.875 ± 0.143	0.0215 ns
VR 0 cm	0.086 ± 0.020	0.067 ± 0.024	<0.001 ***	0.779 ± 0.106	0.851 ± 0.114	<0.001 ***
VR 10 cm	0.082 ± 0.039	0.058 ± 0.039	<0.001 ***	0.824 ± 0.161	0.905 ± 0.139	<0.001 ***
VR 30 cm	0.075 ± 0.034	0.058 ± 0.026	0.0061 ns	0.851 ± 0.107	0.913 ± 0.057	0.0061 ns
VR 50 cm	0.087 ± 0.034	0.070 ± 0.034	0.0046 **	0.819 ± 0.115	0.884 ± 0.086	0.0061 ns

Note. Values are mean ± SD. Pairwise Wilcoxon signed-rank tests (two-tailed), paired by participant. Bonferroni correction applied across 10 simultaneous comparisons per scene (5 conditions × 2 metrics; corrected α = 0.005). ns = not significant after Bonferroni correction; ** *p* < 0.005; *** *p* < 0.001.

**Table 2:** Condition-level Residual Statistics and One-sample Wilcoxon Signed-Rank Test Results.

Scene	Input	Condition	Mean ± SD (N·m·kg^−1^)	Median (N·m·kg^−1^)	W	*p*-Value	rb
Scene 1	Input 1	30 cm	+0.017 ± 0.038	+0.014	25.0	0.1677	+0.451
		VR 0 cm	+0.004 ± 0.047	+0.009	32.0	0.2166	+0.391
		VR 10 cm	+0.001 ± 0.039	−0.005	45.0	10.000	+0.011
		VR 30 cm	+0.000 ± 0.039	+0.004	44.0	0.9460	+0.033
		VR 50 cm	+0.020 ± 0.040	+0.020	21.0	0.0942	+0.538
Scene 1	Input 2	30 cm	−0.009 ± 0.050	−0.020	30.0	0.3054	−0.341
		VR 0 cm	−0.019 ± 0.068	−0.025	37.0	0.3575	−0.295
		VR 10 cm	−0.011 ± 0.079	−0.006	39.0	0.6848	−0.143
		VR 30 cm	−0.019 ± 0.076	−0.002	40.0	0.7354	−0.121
		VR 50 cm	+0.022 ± 0.077	+0.034	33.0	0.4143	+0.275
Scene 2	Input 1	30 cm	+0.010 ± 0.026	+0.006	26.0	0.1909	+0.429
		VR 0 cm	+0.011 ± 0.018	+0.008	20.0	0.0419	+0.619
		VR 10 cm	−0.011 ± 0.031	−0.005	30.0	0.3054	−0.341
		VR 30 cm	−0.002 ± 0.024	+0.000	44.0	0.9460	−0.033
		VR 50 cm	+0.010 ± 0.039	+0.001	34.0	0.4548	+0.253
Scene 2	Input 2	30 cm	+0.010 ± 0.015	+0.015	20.0	0.0803	+0.560
		VR 0 cm	+0.006 ± 0.021	+0.000	48.0	0.8077	+0.086
		VR 10 cm	−0.008 ± 0.012	−0.008	14.0	0.0266	−0.692
		VR 30 cm	+0.001 ± 0.013	+0.006	33.0	0.4143	+0.275
		VR 50 cm	+0.003 ± 0.025	−0.000	39.0	0.6848	+0.143

Note. Residual = y_true − y_pred (positive values indicate model underestimation; negative values indicate model overestimation). Median is reported as it corresponds to the location parameter tested by the Wilcoxon signed-rank test (H_0_: median = 0). W = Wilcoxon signed-rank test statistic. rb = rank-biserial correlation (effect size for one-sample Wilcoxon test). Effect size interpretation: |rb| < 0.10; Small, 0.10 ≤ |rb| < 0.30; Medium, 0.30 ≤ |rb| < 0.50; Large, |rb| ≥ 0.50. Bonferroni correction applied across 5 conditions per scene–input combination (corrected α = 0.010). No condition reached Bonferroni-corrected significance across all four scene–input combinations, indicating an absence of systematic directional bias in model residuals under any VR perturbation condition.

## Data Availability

The datasets generated and analyzed during the current study are available from the corresponding author on reasonable request. The data are not publicly available because they contain human participant biomechanical data and are subject to ethical and privacy restrictions.

## Abbreviations

The following abbreviations are used in this manuscript:

ACL	anterior cruciate ligament
DVJ	drop vertical jump
HMD	head-mounted display
LOCO	leave-one-condition-out
LOSO	leave-one-subject-out
NRMSE	normalized root mean square error
RBF	radial basis function
RMS	root mean square
sEMG	surface electromyography
SVR	support vector regression
VR	virtual reality

## Appendix A

### Appendix A.1

**Table A1:** Participant characteristics (all male, *n* = 14).

Age (years)	Weight (Kg)	Height (cm)	Skeletal Muscle Mass (Kg)	Percent Body Fat (%)	ECW Ratio	Leg Lean Mass (Kg)	Left Achilles Tendon Length (cm)	Right Achilles Tendon Length (cm)	Dominant Side
24	69.9	172	31.6	19.8	0.374	17.8	21	21.3	Left
24	80.4	180	36.6	20	0.372	20.1	22	22	Left
23	70.9	177.2	36.2	10.8	0.369	19	24.5	24.5	Right
22	71.7	172	34.5	15.7	0.366	18.2	20	20	Left
24	74.6	190	37	12.2	0.377	23.1	25	24.5	Left
26	86.6	187	43.8	12.3	0.366	23.6	25	25	Left
25	67.5	174.5	32.7	14.6	0.369	18.2	24	24	Right
20	79.6	174	35.2	22.3	0.37	18.8	23	22	Right
23	88.9	182	46.5	9.9	0.365	23.2	22.5	22.5	Right
22	71.2	169.2	33.1	18.1	0.368	17.3	23	23	Left
19	69.7	177	35.9	10.1	0.364	20.3	23	24	Right
18	65.5	178.5	31.5	13.8	0.38	18.5	24	23.5	Left
18	57.4	173	30.2	7.6	0.364	17.7	22.5	22	Right
22	75.3	177.7	35.3	17	0.373	19.6	23	23	Right

### Appendix A.2

**Table A2:** Phases of Jump Analysis with Criteria and Descriptions.

Phase	Phase Name	Start Criteria	End Criteria	Description
1	Quiet Standing	From the onset of valid data	Right foot potential energy decreases below 95% of peak value during first aerial period (E1)	Preparatory standing phase on the drop box.
2	Step-Off Initiation	From E1	Left foot potential energy drops below 92% of peak value (E2)	Active step-off from the drop box.
3	Aerial Descent	From E2	First initial contact (IC_1_) when foot potential energy drops below 15% of range and ankle force increases rapidly	Descent during the aerial phase. First contact defined by specific criteria for energy and force.
4	Initial Landing Absorption	From IC_1_	First peak knee flexion (E4), detected by peak detection algorithm for knee angles	Eccentric muscle contractions for impact absorption, highest joint loading.
5	Push-Off	From E4	Toe-off (TO_1_), when foot potential energy rises and ankle force approaches zero	Transition from eccentric to concentric contraction, generating vertical force.
6	Aerial Flight	From TO_1_	Second initial contact (IC_2_), pelvis potential energy reaches its peak value (E6)	Flight phase where jump height reaches maximum, with the same IC_2_ criteria as for IC_1_.
7	Secondary Landing Absorption	From IC_2_	Second peak knee flexion (E8), using the same criteria as E4	Absorption of impact on secondary landing, similar to the first landing phase.
8	Stabilization	From E8	Pelvis linear momentum remains below 8% of peak value over 25 frames and pelvis potential energy change is minimal	Final stabilization phase, characterized by steady pelvis momentum and energy.

### Appendix A.3

**Table A3:** Paired Wilcoxon Signed-Rank Test Results Comparing Input2 versus Input1 Across Experimental Conditions Within Each Generalization Scene (Bonferroni-Corrected).

Generalization Scene	Condition	Δr (Input2 − Input1)	W Statistic	*p* Value	Bonferroni sig. (α = 0.010)	rb
Scene 1 (Cross-subject)	30 cm	+0.007	29.0	0.2734 ns	No	+0.363
	VR 0 cm	+0.034	33.0	0.2412 ns	No	+0.371
	VR 10 cm	+0.079	0.0	<0.001 ***	Yes ***	+1.000
	VR 30 cm	+0.010	29.0	0.2734 ns	No	+0.363
	VR 50 cm	−0.001	45.0	1.0000 ns	No	−0.011
Scene 2 (Cross-condition)	30 cm	+0.030	0.0	<0.001 ***	Yes ***	+1.000
	VR 0 cm	+0.038	13.0	0.0107 ns	No	+0.752
	VR 10 cm	+0.012	13.0	0.0215 ns	No	+0.714
	VR 30 cm	+0.028	8.0	0.0061 **	Yes **	+0.824
	VR 50 cm	+0.025	3.0	0.0012 **	Yes **	+0.934

ns, not significant; ** *p* < 0.01; *** *p* < 0.001. Bonferroni significance was determined using the corrected α = 0.010.
